# Convolutional neural network in upper limb functional motion analysis after stroke

**DOI:** 10.7717/peerj.10124

**Published:** 2020-10-09

**Authors:** Agnieszka Szczęsna, Monika Błaszczyszyn, Aleksandra Kawala-Sterniuk

**Affiliations:** 1 Faculty of Automatic Control, Electronics and Computer Science, Silesian University of Technology, Gliwice, Poland; 2 Faculty of Physical Education and Physiotherapy, Opole University of Technology, Opole, Poland; 3 Faculty of Electrical Engineering, Automatic Control and Informatics, Opole University of Technology, Opole, Poland

**Keywords:** Convolutional neural network, Hyperparameters, Functional motion analysis, Stroke, Lifting movements, Optical motion capture

## Abstract

In this work, implementation of Convolutional Neural Network (CNN) for the purpose of analysis of functional upper limb movement pattern was applied. The main aim of the study was to compare motion of selected activities of daily living of participants after stroke with the healthy ones (in similar age). The optical, marker-based motion capture system was applied for the purpose of data acquisition. There were some attempts made in order to find the existing differences in the motion pattern of the upper limb. For this purpose, the motion features of dominant and non-dominant upper limb of healthy participants were compared with motion features of paresis and non-paresis upper limbs of participants after stroke. On the basis of the newly collected data set, a new CNN application was presented to the classification of motion data in two different class label configurations. Analyzing individual segments of the upper body, it turned out that the arm was the most sensitive segment for capturing changes in the trajectory of the lifting movements of objects.

## Introduction

The human pose tracking is also known as motion capture (MoCap) and has been studied for decades and is still a very active and challenging research topic, (Xiao, Wu & Wei, 2018; Ganapathi et al., 2012). Currently a specialized computer vision and marker-based MoCap technique, called Optical Motion Capture is being used and constitutes the gold-standard for accurate and robust motion capture, (Guerra-Filho, 2005; Merriaux et al., 2017; Samy et al., 2020).

The Artificial Neural Networks (ANNs) are powerful tools and they are more and more frequently used to analyze motion data. It has been proven that ANN is effective tool for kinematics analysis, (Gomi & Kawato 1993; Meadmore et al. 2014). Kinematic analysis is the study of the motion of the body, limbs, and joints, which occurs during movement. This method of analysis provides a non-invasive means of collecting objective information on joint and limb motion from humans.

The captured motion data is used in many analyses, such as inter alia: classification of motion capture human gait data (Switonski, Josinski & Wojciechowski, 2019; Szczesna et al., 2018), identifying the presence of deterministic chaos in motion capture human gait data (Piorek et al., 2017; Szczęsna, 2019), template generation and comparing based on motion capture data of karate athletes (Hachaj, Piekarczyk & Ogiela, 2017).

This work concerns analysis of upper limb motion data of patients after stroke. The traditional methods used for the upper limb rehabilitation assessment purposes usually consisted of paper functional scales, such as e.g., Fugel-Meyer (FMA), Wolf Motor Function Test (WMFT), Action Research Arm Test (ARAT), (Rech et al., 2020; Turtle, Porter-Armstrong & Stinson, 2020). These are unfortunately subjective tools. The studies provided evidence that upper limb kinematics change after stroke compared to the control group. Kinematic differences between participants with and without stroke showed consistent patterns despite the variety of tasks and participants. The meta-analysis (Collins et al., 2018) shows that individuals with stroke perform reach-to-grasp tasks with lower peak velocity, longer movement time, decreased movement smoothness, increased curvature of reach path ratio, greater trunk displacement and less elbow extension than control participants. All these kinematic features are therefore potential clinical goals of rehabilitation therapy. The kinematic differences between participants with stroke and control remain constant during reach-to-grasp in the central and ipsilateral workspace for velocity and movement time.

Smoothness, jerk, speed, trajectory of upper limbs are used as criteria for the rehabilitation status of upper limbs assessment. In addition, fluidity and continuity of upper limb movements are also considered, as they can reflect the current functional status of patients and assess the progress of ongoing rehabilitation in an objective manner. For these reasons, researchers are developing new methods and technologies in order to make the rehabilitation process more effective, faster and more convenient for the patient (Goffredo et al., 2008).

The main purpose of this study was application of the Convolutional Neural Network (CNN) for upper limb functional motion analysis during selected ADLs (activities of daily living) based on this same motion acquisition procedure as in previous work (Blaszczyszyn et al., 2018). This is new application of CNN for classification of motion data. Based on classification accuracy results in two classification tasks, the most important skeleton segments (based on marker position) in classification of upper limb functional motion were defined.

### Related work

The class of ANN covers several network architectures including Convolutional Neural Networks (CNNs). The CNNs due to implementation of local connectivity patterns efficiently with shared weights, have quickly become a state-of-the-art method for image, video, and natural language processing etc. They are even frequently applied for the purpose of biomedical signals’ processing such as inter alia ECG, (Xu & Liu 2020).

The CNN enable local patterns recognition and is used in order to identify human actions from the temporal variation of these features, which are distorted due to the inconsistency in the execution of actions across observations and subjects, (Ijjina & Mohan, 2014).

Research presented by Guo, Gok & Sahin (2018) focused on implementation of the CNN model for prediction of the short-term dynamics of qualified forelimb movements based on neural signals in multiple animals. In Panwar et al. (2019), the effective classification of three upper arm movements is presented. The ANN methods were used to assess rehabilitation based on Cao et al. (2019), assess the progress of rehabilitation under the influence of a computer game (Bai, Song & Li, 2019) and analysis of the myoelectric signal during movement of upper limbs (Mukhopadhyay & Samui, 2020).

The CNNs achieved an almost perfect classification on physical activities, especially very similar ones which were previously perceived to be very difficult to classify. The CNNs outperform other state-of-the-art data mining techniques in HAR (Human Activity Recognition) (Ronao & Cho, 2016; Cho & Yoon, 2018).

The CNN is very efficient in image recognition, in which local spatial dependencies do exist, (Islam, Wijewickrema & OLeary, 2019). The same advantage can be usefully in motion analysis where the input data is organized into array represented with some snapshots of motion in form of activity image (Liu et al., 2016; Ijjina & Mohan, 2014).

Recently, CNNs have emerged as the 3D full-body human pose estimation from a marker-less monocular RGB (Red-Green-Blue) image sequence, (Zhou et al., 2018). In Ma, Sun & Sun (2018), the cascade CNN was used to upper limb estimation of joints position based on RGB-D quad channels image (color and depth information). These two approaches prove the usefulness of the CNNs in motion data analysis but are intended for a marker-less motion data acquisition systems. In the proposed application, the input is data from an optical marker-based system.

This work presents application of the general motion patterns, which are an upper limb lifting movements. The carried out analysis concerns some functional differences in motion. The thorough literature studies have proven that upper limb kinematics change as a result of stroke in comparison to the healthy ones from the control participants. The differences identified between individuals affected with stroke and those healthy can be used to advance interventions targeted at the underlying movement deficits. One way of improving our understanding of the time course of recovery may be to proceed by investigating the factors, which allow to predict the pattern of functional recovery as a function of time. The way the stroke affected patients try to control their functional movements is consistent in accordance with the Bernstein’s theory of human movement behavior. The most fundamental solution to the problem of controlling co-ordination functional movements consists of reducing the number of independent elements to be controlled. These changes enable patients to control functional tasks with more accuracy and less energy by reducing the number of degrees of freedom (Latash & Anson, 1996; Kwakkel, Kollen & Lindeman, 2004; Collins et al., 2018).

## Materials & Methods

Motion data are markers placed on human body segments motion trajectories in 3D space captured by an optical motion data acquisition system. The performed analysis concerns the generalization of upper limb lifting movements in healthy elderly participants and post-stroke patients. The data was recorded during each hand lifting (*left* and *right*) to the head height of Small Cylinder (SC) and Large Cylinder (LC). It also involved analysis of Drinking Operation (DG), where the glass lifting has also been performed, although only to the mouth level.

The main task was to check the most significant body segment in kinematic chain for classification accuracy. The main aim of this work involved two tasks defined for the CNN. One of them was classification to distinguishing between types of upper limb (3 classes) and second one to differentiate patients’ condition (2 classes) upon his/her upper limb movements. This study is based on already collected data.

During the studies—two types of problem were considered, based on the collected data:

1. upper limb class recognition (three classes: healthy participant’s upper limb / paresis participant’s upper limb after stroke / non-paresis upper limb of participant after stroke);
2. recognition of the participant’s class (two classes: healthy participant’s upper limb / upper limb of the participant after the stroke).

Suitable for the carried out tasks CNN models were proposed and the prepossessing of data and features’ calculation was described in this work. Also the hyperparamterization of the applied model was presented. Based on the obtained results, the segments of the kinematic chain (skeleton), which were most relevant to the specific classification task, were identified.

The captured motion trajectory signals represented a kinematic chain of the upper limb (a set of markers placed on upper limb segments). Based on that the first defined task concerns possibility to identify the most sensitive segments for the trajectory disturbances of the lifting movement of upper limb. The aim of the second task of the carried out experiments was to show bilateral disorder in people after stroke, this is in line with the theory, which assumes that after damage to certain areas of the brain it is possible to notice some changes in motor functions also in non-affected areas (side). The obtained results confirmed the need for application of bilateral therapies.

### Conducted experiments

A total number of 54 participants were recruited to this study, including 35 patients of the stroke group (G1) and 19 healthy subjects (control group, G2).

Among the 35 affected with the ischemic stroke participants there were 16 women and 19 men (mean age 67 ± 8.9 years). These patients were 3–16 months after the first stroke, 20 of them were with right-hand paresis and 15 with left-hand paresis.

The control group (G2) consisted of 19 recruited healthy participants, whose age-matched the post-stroke patients and included 14 women and 5 men (mean age: 64 ± 9.0 years).

The below criteria were met for the stroke group of participants (G1): spasticity ≤2 in accordance with the modified Ashworth scale, who were able to stretch the affected arms out; no apraxia or shoulder pain that may interfere with task accomplishment; no any neuromuscular or orthopaedic disorders or major visual attention problems, major perceptual or cognitive deficits and ability to provide informed consent, disturbances of cognitive functions measured by Mini–Mental State Examination (MMSE ≥ 24); NIHSS (National Institutes of Health Stroke Scale) total score: median 4 (2 –5); Fugl-Meyer Motor Assessment (FMA –upper limb ): 44 ± 8.2.

All participants have received detailed information regarding the experimental procedure, which was going to be carried out and all of them gave their written consent for the study participation. The research project’s protocol was approved by the Bioethics Committee at the Opole Medical Chamber No. 215, on 25th March 2015 and the study was performed in accordance with the Helsinki Declaration’s recommendations for clinical trials on humans.

### Motion data acquisition

For the purpose of 3D trajectory segments acquisition the optical motion analysis system based on passive, reflective markers with eight infrared cameras, was used (OptiTrack system, NaturalPoint, Inc. https://optitrack.com/). The motion acquisition frequency was 100 Hz.

The procedure was the same as in Blaszczyszyn et al. (2018) where only forward and back transporting phases of a cylinder movement were taken into account. The third performed task was drinking, where the first phase was to reach out for the glass from the starting position and then to grasp and move the glass forward to the mouth to drink and to place it back to the the table behind a marked line; and finally—to return to the initial position in accordance with the drinking phase definition in Murphy, Willén & Sunnerhagen (2011).

**Figure 1:**
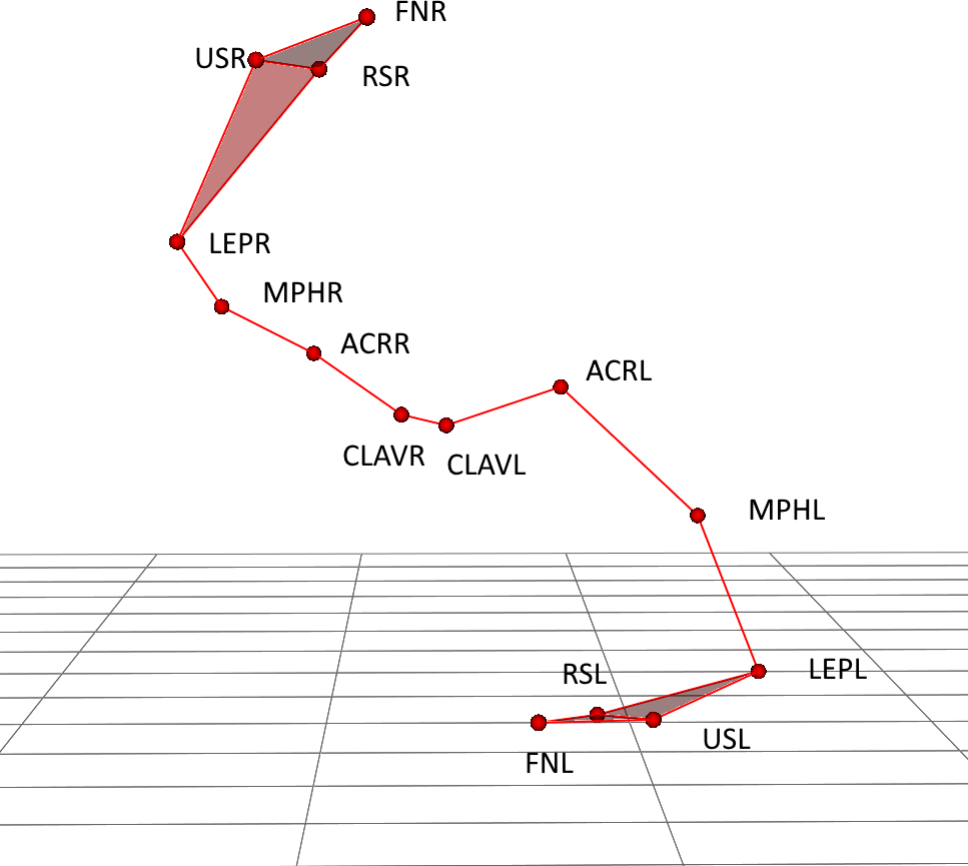
Upper body marker schema during lifting the large cylinder with right hand.

The markers were located on both upper limbs (right –R and left –L) on the following locations: clavicular sternal (CLAVR/L), acromion process (ACRR/L), middle part of the humeri (MPHR/L), lateral epicondyle (LEPR/L), radial styloid (RSR/L), ulnar styloid (USR/L), index finger nails (FNR/L) (Fig. 1). For presented application only trajectory of four markers ACRR/L (shoulder segment), MPHR/L (arm segment), LEPR/L (forearm segment) and FNR/L (hand segment) was applied.

The protocol describes following functional activities as follows: drinking from glass (DG), lifting a small cylinder (SC), lifting a large cylinder (LC).

The motion activities were measured with the OptiTrack system during the above defined tasks. The presented analysis was based on the 3D trajectory of the markers placed on the upper limb skeleton segments.

### Convolutional neural network model

The CNNs are a specialized kind of neural network for processing data that has a known, grid-like topology. Classification using grid-like topology in case of motion data allows to process whole motion (lifting and lowering the hand) without split into windows. Also the motion of skeleton segments (markers) are treated in a correlated way. The proposed solution is a new application of the CNN based on the collected motion data of upper limb movement.

The convolutional layers used in the network are characterized by the learning of local patterns. This behavior makes these networks to have two key properties of the convolution networks: it allows to find patterns, regardless of their displacement and distortion; it has the ability to create a hierarchy of patterns. They take advantage of the hierarchical pattern in data and assemble more complex patterns using smaller and simpler patterns learned by previous layers.

This paper presents a solution for analysis of human functional motion during ADLs based on the implementation of the CNNs, which allows local patterns recognition by translation of invariance characteristics. Due to the fact that the position and scale of learned pattern is not important—it allows to find differences in movement pattern of the upper limb lifting movement.

**Figure 2:**
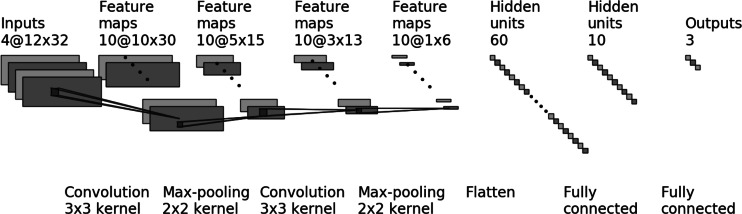
The CNN model for four markers (four input layers) and three output classes.

The proposed CNN model consists of the following layers (Fig. 2) with the following hyperparameters:

• First convolution layer, where the input samples were processed using set numbers of filters (hyperparameter *f*_*Conv*1_). The convolution layers used in the proposed network use a 3 × 3 kernel. The Rectified Linear Unit (ReLU) was used as an activation function.
• In the next stage, the processed data goes to the scaling layer, which performs the max-pooling operation with 2 × 2 window size.
• The third layer is convolution layer with set of filters (hyperparameter *f*_*Conv*2_) and 3 × 3 kernel. The Rectified Linear Unit (ReLU) is used as an activation function.
• In the next stage, the processed data goes to the scaling layer, which performs the *max-pooling* operation with 2 × 2 window size.
• Next is a flattening layer.
• Dense layer with the Rectified Linear Unit (ReLU) used as activation function.
• Next is a dropout layer. This layer is applied between the last hidden layer and the output layer. The dropout rate is hyperparameter *d*_*Drop*1_.
• Second dense layer with 3 or 2 outputs (depends on numbers of output classes). The activation function is softmax.

The Rectified Linear Unit (ReLU) is used as the activation function for hidden layers as it is default recommendation for CNN in (Goodfellow, Bengio & Courville, 2016). The ReLU function overcomes the vanishing gradient problem, allowing models to learn faster and perform more efficiently.

Classification accuracy is the total number of correct predictions divided by the total number of predictions made for a data-set. It is so, because for presented class definition unbalance occurs the AUC (area under the receiver operating characteristic curve) metric was used as a performance measure.

For the training purpose of the proposed CNN model the Adam optimizer were used. The loss value, which will be minimized by the model, was multi-class cross-entropy.

### Data prepossessing

The Table 1 presents the summary of all input data with numbers of recordings (input data files). One recording consists of one functional motion (for example lifting and lowering the large cylinder). The G1 is group of participants after stroke and G2 is a control group of elderly participants. Dataset was splitted into training and validation set in ratio 80% to 20%. The given results are evaluation results of the validation data.

During the study, two classification tasks were proposed and tested. The first one was based on the upper limb type with the three following classes:

• *HUL* (Healthy Upper Limb) for upper limb of G2 participants (healthy control group), and contains all activities performed by participants form G2 (114 input data sets);
• *NPUL* (Non-Paretic Upper Limb) for non-paresis upper limb of G1 participants, contains activities performed by participants from G1 with non-paresis limb (105 input data sets);
• *PUL* (Paretic Upper Limb) for paresis upper limb of G1 group, contains of activities performed by paresis limb are labeled as class *PUL* (105 input data sets).

Second classification task is based on type of participant group, where:

• class *G1* is for all data sets of participants after stroke in G1, regardless of the type (paresis/non paresis) of arm (210 input data sets);
• class *G2* contains all data sets (left and right upper limb) of participants form control group G2 (114 input data sets).

Each plane (XY, YZ, XZ) of motion was analyzed separately, the input signal is the coordinate value in 3D space. The data prepossessing steps are following:

**Table 1:** Numbers of recordings (input data sets). G1 is group of participants after stroke, G2 is a control group of elderly participants.

**Participants group**	**Upper limb**	**Activity**		
		LC	SC	DG
G1	non-paresis	35	35	35
	paresis	35	35	35
G2	left	19	19	19
	right	19	19	19

**Figure 3:**
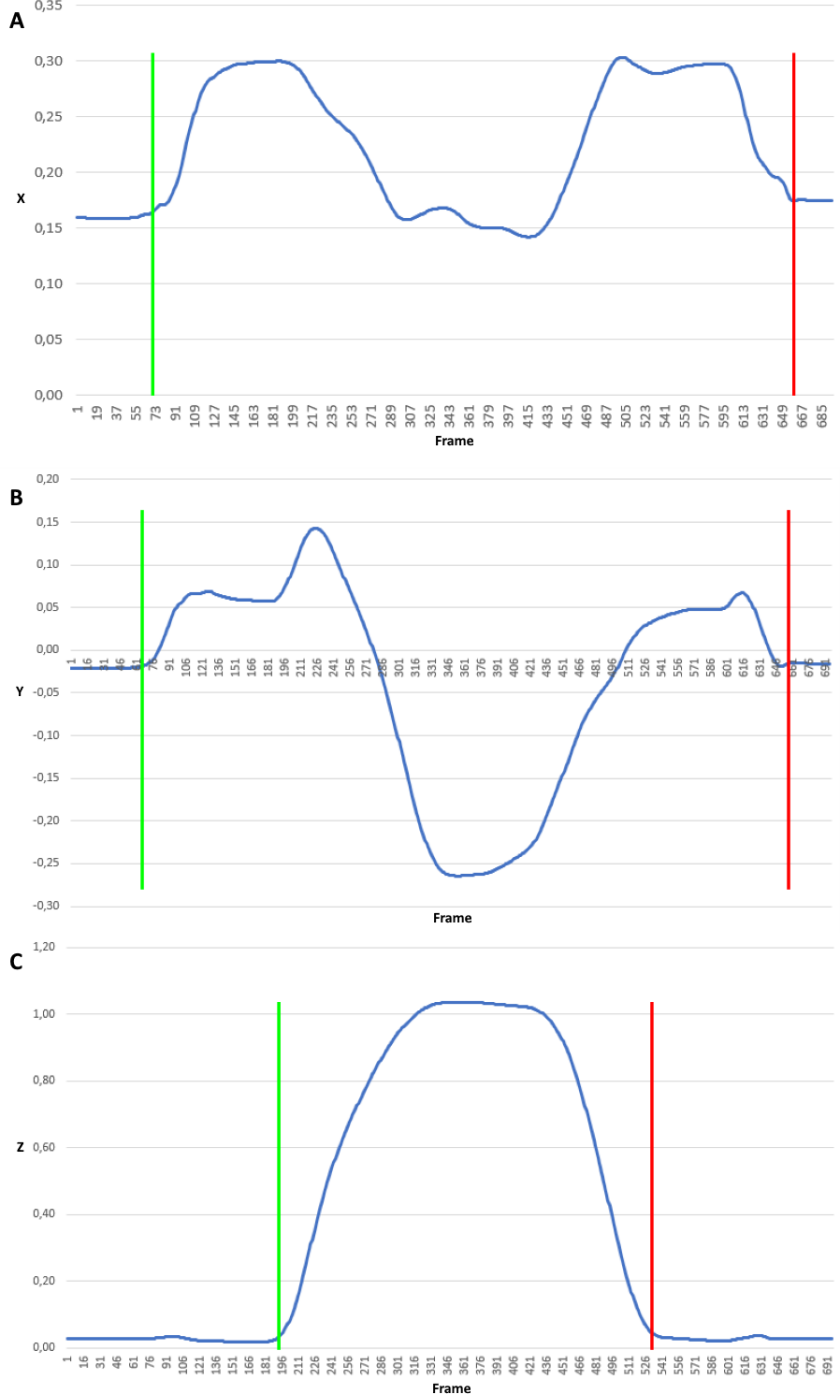
Trajectory coordinates of the FNL marker when lifting a small cylinder. Signal clipped from the beginning of the movement (green border) to the end (red border) for each plane independently: (A) X coordinate, (B) Y coordinate, (C) Z coordinate.

• Cut out the signal represented coordinates regarding movement in every plane. Rejection of insignificant data from the beginning and the end of signals (Fig. 3). In Fig. 3 positions of the FNL marker (marker on left index finger) when lifting a small cylinder were presented. It illustrates the coordinate signal clipped from the beginning of the movement (marked with green border) to the end (marked with red border) for each plane independently.
• Unification of the motion side, all left limb movement signals has been transformed (reflected) to the form of right limb movement. After this unification, the marker symbols are given without the last letter denoting the body side.
• Resampling of the input signals in meaning of change the sample rate. Regardless of the signal length (duration), trajectory signals have been resampled to fixed numbers of samples by linear interpolation. The predefined samples number was set to 32.
• Based on clipped coordinate signal consisting of *N* + 1 samples, *p*_*k*_ = (*p*_*x*,*k*_, *p*_*y*,*k*_, *p*_*z*,*k*_), *k* ∈ [0, *N*], features based on 3D trajectory in each plane are determined: displacement (*d*_*x*_, *d*_*y*_, *d*_*z*_), velocity (*v*_*x*_, *v*_*y*_, *v*_*z*_), acceleration (*a*_*x*_, *a*_*y*_, *a*_*z*_) and jerk (*j*_*x*_, *j*_*y*_, *j*_*z*_). Displacement is the Euclidean distance between the subsequent markers positions for each dimension *d*_*x*|*y*|*z*,*i*_ = |*p*_*x*|*y*|*z*,*i*_ − *p*_*x*|*y*|*z*,*i*−1_|. Velocity feature is vector of temporal velocities ${v}_{x{|}y{|}z,i}= \frac{{d}_{x{|}y{|}z,i}-{d}_{x{|}y{|}z,i-1}}{T} $, where *T* is sampling interval. Similarly the acceleration feature is obtained ${a}_{x{|}y{|}z,i}= \frac{{v}_{x{|}y{|}z,i}-{v}_{x{|}y{|}z,i-1}}{T} $. Jerk is the rate of acceleration changes with respect to time. Jerk can be expressed as the first time derivative of acceleration, second time derivative of velocity, and third time derivative of position ${j}_{x{|}y{|}z,i}= \frac{{a}_{x{|}y{|}z,i}-{a}_{x{|}y{|}z,i-1}}{T} $, where *i* ∈ [1, *N*].
• Input data normalization by *min-max* normalization to $ \left[ 0,1 \right] $ range using following equitation ${f}^{{^{\prime}}}= \frac{f-\min }{\max -\min } $, where *f*′ is normalized feature value *f*.

After cutting out the motion data from the raw input signal, being in the form of trajectory coordinates in three-dimensional space, the values of features (*d*_*x*_, *d*_*y*_, *d*_*z*_, *v*_*x*_, *v*_*y*_, *v*_*z*_, *a*_*x*_, *a*_*y*_, *a*_*z*_, *j*_*x*_, *j*_*y*_, *j*_*z*_) were calculated. Then they were converted to a multidimensional representation, depending on the number of markers used, but with fixed width (predefined 32 samples), height (4 features for each plane gives 12). The depth is based on the number of markers used.

**Figure 4:**
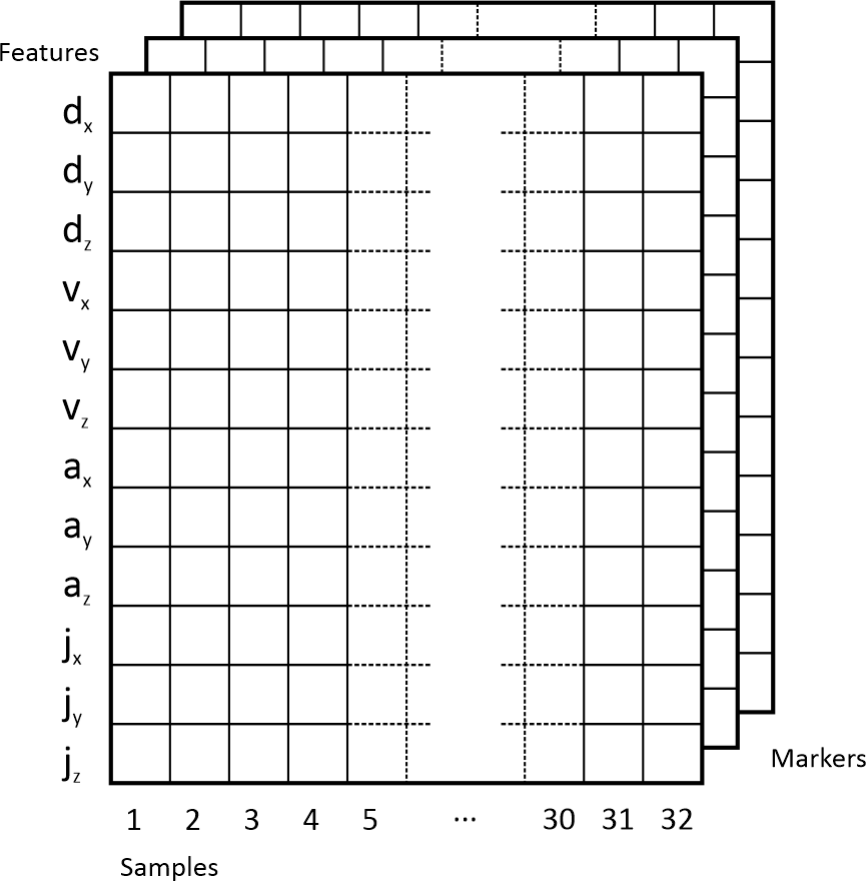
Input data format.

The obtained features were arranged in an array, where the rows represented one feature and the columns contain next time feature samples. The obtained data is a result of calculations for separate markers, which were placed in subsequent layers of this array. The following set of 4 markers placed on upper limb was used in experiments: FN, LEP, MPH and ACR. The names after unification of side are without (L/R). The input data format was illustrated with the Fig. 4.

**Figure 5:**
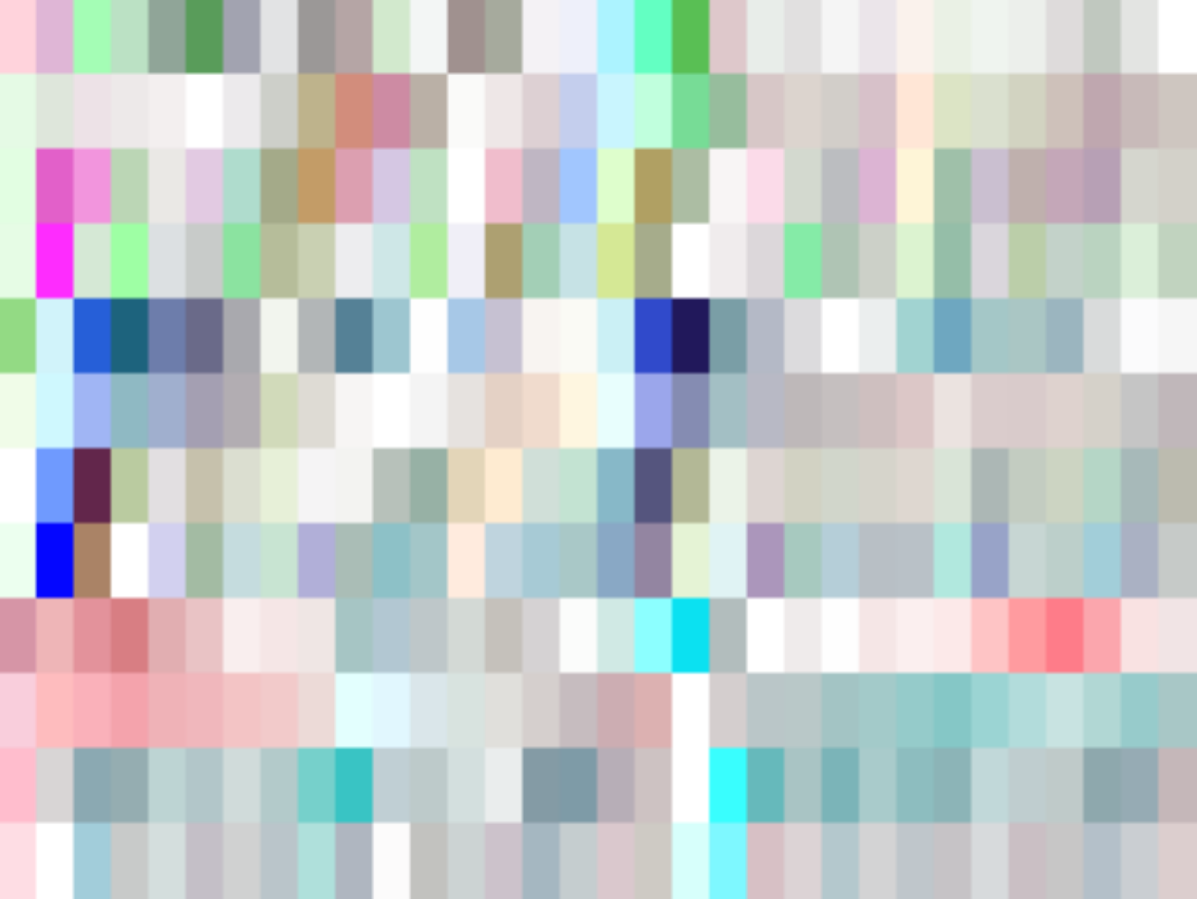
RGBA image (32 × 12 × 4) represents the FN (red channel), LEP (green channel), MPH (blue channel) and ACR (alpha channel) marker data of participant form G1 group.

**Figure 6:**
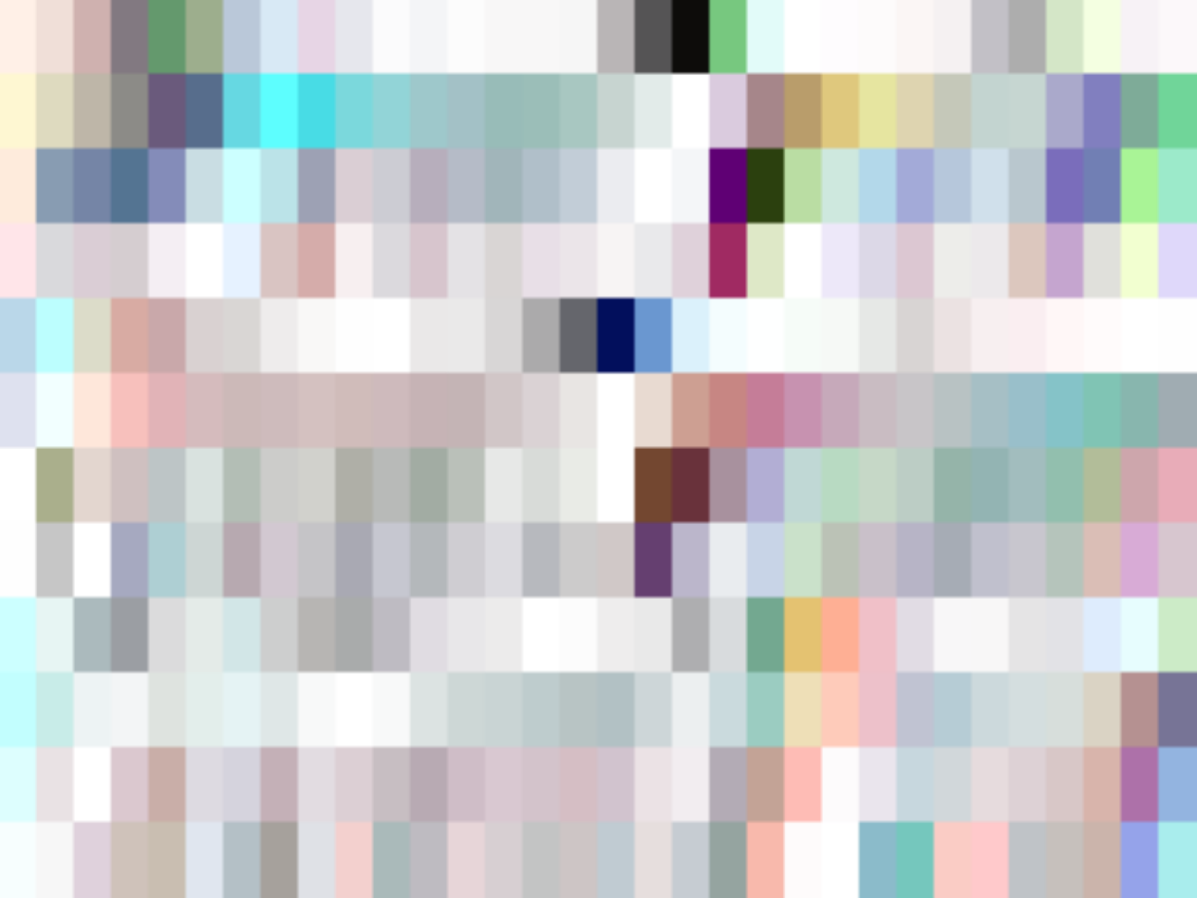
RGBA image (32 × 12 × 4) represents the FN (red channel), LEP (green channel), MPH (blue channel) and ACR (alpha channel) marker data of participant form G2 group.

This representation of the data could be seen as an image, respectively: grey scale image (one channel represents 1 marker), two channel image (two channels for 2 markers), three channel image (RGB image, three channels for 3 markers), four channel image (RGBA image, four channels for 4 markers, Figs. 5 and 6).

The obtained data is based on four markers representing four segments of upper limb: hand (FN), forearm (LEP), arm (ACR) and shoulder (ACR). Such data allows to carry out analysis regarding which segment or segment configuration gives better classification results. Such sensitive location analysis is based on results’ accuracy concerning markers configuration. Firstly only one marker data was used, where depth of input array equals one (grey scale image). Next based on the best results the model obtained from two markers (depth equals 2), three (RGB image) and four markers (RGBA image) were tested.

Hyperparameter optimization finds a tuple of hyperparameters yielding an optimal model which minimizes a predefined loss function on the given data. In the proposed application of the CNN model the following set of hyperparametres was defined: learning rate (*lr*), number of epochs to train the model (*ep*), number of filters used in first convolution layer (*f*_*Conv*1_), number of filters used in second convolution layer (*f*_*Conv*2_), the dropout rate of dropout layer (*d*_*Drop*1_), the batch size of the output space for first dense layer (*out*_*Dense*1_).

Bayesian optimization using Gaussian processes was applied in order to find values of defined hyperparameters to obtain model with the highest metric AUC value on the validation set. Number of calls to find the minimum of (1 − *AUC*) was set to 40. Optimization was run for each experiment independently.

### Implementation

For the development purposes the authors decided to use the *Microsoft Visual Studio 2019*. For the implementation *Python 3.7* (Millman & Aivazis, 2011) was applied with the following most important libraries: *Kreas* as a high-level neural networks programming interface with *TensorFlow* backend (Erickson et al., 2017; Gulli & Pal, 2017 and Chollet, 2015); *Scikit-Optimize* for hyperparameters optimization; *NumPy* for the input data structure preparation as a multidimensional array (Van Der Walt, Colbert & Varoquaux, 2011 and Oliphant, 2006).

## Results

With the implementation of the CNN the following research scheme was planned. The first goal was to find the appropriate marker configuration, which had the greatest impact on the classification of the healthy participants’ upper limb movement (group G2) and those affected with stroke (group G1). Initially the authors of this work proposed division of data into three classes (*HUL*, *NPUL*, *PUL*) based on upper limb type. The next step covered division into classes (*G1*, *G2*), which was proposed based on the participants group and not on the upper limb type (condition). Based on the conguration with the best accuracy, a combination of markers was proposed, which enabled significant improvement of the nal results (Table 2).

The classification applied in both tasks gives satisfactory results. In the two-class problem the higher accuracy was obtained, which is partly due to impact of imbalance of class labels. Therefore, precision and recall are also greater. The most important conclusion is that on the basis of the collected data divided into two classes by type of participant (G1/G2) obtained classification results mostly above 90%.

Classification results for the same defined tasks have also been checked for the classifiers: Random Forest (*RF*) with 100 trees in the forest and maximum depth of the tree equals 10 (Breiman, 2001), Linear Support Vector (*LSV*) (Boser, Guyon & Vapnik, 1992) and Logistic Regression (*LR*) (Bishop, 2006). In general, classifiers had an average accuracy *KF* of 9.8%, *LSV* of 8.4% and *LR* of 7.5% lower in a classification with three classes and *KF* of 5.9%, *LSV* of 13.4% and *LR* of 8.75% lower for two classes. Similar relationships in the classification results for different segments in the participant type classification (G1/G2) were noticeable, but not so pronounced. The best results were for *LR* for four segments configuration FN, LEP, MPH and ACR in three class task - 86%. For two classes the best results were obtained for *RF* for two segment configuration LEP and MPH - 99%. Detailed results comparison was presented in Table 3.

**Table 2:** Hyperparameters optimizations results.

**Markers**	**Upper limb type (three classes)**		**Participants group (two classes)**	
	Hyperparameters	CNN metrices	Hyperparameters	CNN metrices
FN	*lr* = 0.00017		*lr* = 0.0011	
	*ep* = 100	*AUC* = 85.54%	*ep* = 224	*AUC* = 94.53%
	*f*_*Conv*1_ = 77	*Accuracy* = 69.23%	*f*_*Conv*1_ = 83	*Accuracy* = 88.5%
	*f*_*Conv*2_ = 10	*Precision* = 20%	*f*_*Conv*2_ = 100	*Precision* = 88.5%
	*d*_*Drop*1_ = 0.25	*Recall* = 65.38%	*d*_*Drop*1_ = 0.2484	*Recall* = 88.5%
	*out*_*Dense*1_ = 100		*out*_*Dense*1_ = 30	
LEP	*lr* = 0.00037		*lr* = 0.00026	
	*ep* = 100	*AUC* = 89.53%	*ep* = 216	*AUC* = 93.62%
	*f*_*Conv*1_ = 20	*Accuracy* = 80.77%	*f*_*Conv*1_ = 51	*Accuracy* = 92.6%
	*f*_*Conv*2_ = 10	*Precision* = 80.77%	*f*_*Conv*2_ = 10	*Precision* = 92.6%
	*d*_*Drop*1_ = 0.1931	*Recall* = 80.77%	*d*_*Drop*1_ = 0.0638	*Recall* = 92.6%
	*out*_*Dense*1_ = 72		*out*_*Dense*1_ = 11	
MPH	*lr* = 0.00023		*lr* = 0.00004	
	*ep* = 100	*AUC* = 96.18%	*ep* = 261	*AUC* = 99.5%
	*f*_*Conv*1_ = 100	*Accuracy* = 87.50%	*f*_*Conv*1_ = 87	*Accuracy* = 99%
	*f*_*Conv*2_ = 79	*Precision* = 90.91%	*f*_*Conv*2_ = 51	*Precision* = 98%
	*d*_*Drop*1_ = 0.25	*Recall* = 83.33%	*d*_*Drop*1_ = 0.1054	*Recall* = 98%
	*out*_*Dense*1_ = 44		*out*_*Dense*1_ = 19	
ACR	*lr* = 0.01		*lr* = 0.0026	
	*ep* = 300	*AUC* = 83.98%	*ep* = 202	*AUC* = 99.20%
	*f*_*Conv*1_ = 12	*Accuracy* = 58.33%	*f*_*Conv*1_ = 50	*Accuracy* = 96%
	*f*_*Conv*2_ = 54	*Precision* = 60.87%	*f*_*Conv*2_ = 100	*Precision* = 96%
	*d*_*Drop*1_ = 0.004	*Recall* = 58.33%	*d*_*Drop*1_ = 0.2435	*Recall* = 96%
	*out*_*Dense*1_ = 10		*out*_*Dense*1_ = 26	
LEP, MPH	*lr* = 0.00006		*lr* = 0.0064	
	*ep* = 300	*AUC* = 94.62%	*ep* = 300	*AUC* = 99.5%
	*f*_*Conv*1_ = 75	*Accuracy* = 87.5%	*f*_*Conv*1_ = 100	*Accuracy* = 99%
	*f*_*Conv*2_ = 26	*Precision* = 89.47%	*f*_*Conv*2_ = 10	*Precision* = 98%
	*d*_*Drop*1_ = 0	*Recall* = 70.83%	*d*_*Drop*1_ = 0.1797	*Recall* = 98%
	*out*_*Dense*1_ = 98		*out*_*Dense*1_ = 10	
FN, LEP, MPH	*lr* = 0.00012		*lr* = 0.00003	
	*ep* = 300	*AUC* = 90%	*ep* = 300	*AUC* = 98.4%
	*f*_*Conv*1_ = 100	*Accuracy* = 72%	*f*_*Conv*1_ = 95	*Accuracy* = 92%
	*f*_*Conv*2_ = 10	*Precision* = 75%	*f*_*Conv*2_ = 10	*Precision* = 92%
	*d*_*Drop*1_ = 0	*Recall* = 72%	*d*_*Drop*1_ = 0.25	*Recall* = 92%
	*out*_*Dense*1_ = 10		*out*_*Dense*1_ = 100	
LEP, MPH, ACR	*lr* = 0.00005		*lr* = 0.00017	
	*ep* = 296	*AUC* = 83.11%	*ep* = 164	*AUC* = 89.41%
	*f*_*Conv*1_ = 62	*Accuracy* = 68%	*f*_*Conv*1_ = 100	*Accuracy* = 86.9%
	*f*_*Conv*2_ = 87	*Precision* = 68.11%	*f*_*Conv*2_ = 78	*Precision* = 86.9%
	*d*_*Drop*1_ = 0.0845	*Recall* = 68.11%	*d*_*Drop*1_ = 0.0126	*Recall* = 86.9%
	*out*_*Dense*1_ = 72		*out*_*Dense*1_ = 85	
FN, LEP, MPH, ACR	*lr* = 0.00019		*lr* = 0.000074	
	*ep* = 300	*AUC* = 96.33%	*ep* = 290	*AUC* = 99.7%
	*f*_*Conv*1_ = 10	*Accuracy* = 77.27%	*f*_*Conv*1_ = 22	*Accuracy* = 99%
	*f*_*Conv*2_ = 65	*Precision* = 77.27%	*f*_*Conv*2_ = 39	*Precision* = 98.5%
	*d*_*Drop*1_ = 0.25	*Recall* = 77.27%	*d*_*Drop*1_ = 0.2491	*Recall* = 98.5%
	*out*_*Dense*1_ = 100		*out*_*Dense*1_ = 99	

**Table 3:** Classification accuracy results for classifiers: Random Forest (RF), Linear Support Vector (LSV) and Logistic Regression (LR).

**Markers**	**Upper limb type (three classes)**	**Participants group (two classes)**
FN	*RF* = 65%	*RF* = 88%
	*LSV* = 65%	*LSV* = 77%
	*LR* = 60%	*LR* = 88%
LEP	*RF* = 61%	*RF* = 89%
	*LSV* = 54%	*LSV* = 85%
	*LR* = 61%	*LR* = 85%
MPH	*RF* = 67%	*RF* = 96%
	*LSV* = 62.5%	*LSV* = 79%
	*LR* = 67%	*LR* = 83%
ACR	*RF* = 62.5%	*RF* = 80%
	*LSV* = 67%	*LSV* = 80%
	*LR* = 62.5%	*LR* = 84%
LEP, MPH	*RF* = 71%	*RF* = 99%
	*LSV* = 71%	*LSV* = 83%
	*LR* = 71%	*LR* = 92%
FN, LEP, MPH	*RF* = 60%	*RF* = 92%
	*LSV* = 60%	*LSV* = 84%
	*LR* = 60%	*LR* = 84%
LEP, MPH, ACR	*RF* = 68%	*RF* = 74%
	*LSV* = 77%	*LSV* = 83%
	*LR* = 73%	*LR* = 83%
FN, LEP, MPH, ACR	*RF* = 68%	*RF* = 91%
	*LSV* = 77%	*LSV* = 78%
	*LR* = 86%	*LR* = 87%

The carried out studies concerned the configuration of markers for the upper limb model. It was attempted to identify the most sensitive locations of the movement trajectory disorders during lifting objects. The first adopted hypothesis assumed the activity of the distal part of limb. But the obtained results proved that the marker on the finger (marker FN) and forearm segment (marker LEP) were not sensitive enough, here in two class classification accuracy was 88.5% (FN) and 92.6% (LEP). Therefore a second hypothesis was made concerning proximal limb parts with marker placed on the shoulder (marker ACR) and in the middle of the arm (marker MPH). In this case the gathered results were satisfying for the marker located in the middle of the arm (marker MPH, accuracy about 99%) and with combinations with forearm (LEP, MPH, accuracy about 99%) and all upper limb markers (FN, LEP, MPH, ACR, accuracy about 99%). The same trend was visible in two classification variant based on upper limb type and participants group. The definite and worst results were achieved for movement of shoulder (ACR marker) in upper limb type classification (accuracy 58.33% in three classes classification). In classification of type of participant the worst results were for finger (marker FN) with accuracy about 88.5%.

## Discussion

This type of research can contribute to the development of an objective tool for qualification of the patient for rehabilitation and gives the opportunity to monitor rehabilitation progress and also enables to program task-based rehabilitation (Bai, Song & Li, 2019). On the other hand it contributes to the identification of the most sensitive places in the kinematic chain, which are the most suitable for sensors placement.

The obtained results show that the most prone to detection of the trajectory disturbances body segment is arm. Thus, it proved that the proximal body segment and not the distal one turned out to be the most sensitive to the studied changes. This may seem to contrary to the results of studies, which prove that the hand movement and the gripping function are the most forecasting for prediction of the functional state after stroke.

The second aspect of the carried out study concerned bilateral activities. The proposed CNNs model for classification of upper limb movement based on participant type (healthy/after stroke) gives very good results. So that the proposed features strongly differentiate the movement between G1 and G2 group, without distinguishing the affected/not affected side for stroke participants (G1).

Therefore, despite maintaining the correct anatomical structure of movement, the general condition of movement patterns changes after stroke. The bilateral movements are required in many daily activities and, as observations showed, bilateral training can be more benecial for the daily activities for people after stroke (Lee et al., 2017). Motor changes in the non-paresis limb are not manifested in parameters commonly assessed with the clinical scales, such as mobility ranges. But they are visible in the spatio-temporal parameters of the assessed movement, which are difficult to capture during visual assessment. They require usage of additional equipment and analysis. These include used in that study features like: jerk, acceleration, velocity and traveled distance of body segments during the movement.

## Conclusions

The study on the CNN model applied for the purpose of upper limb kinematics analysis can give accurate and objective information regarding human movement and is therefore a powerful tool for both clinical and research domains.

In this paper—the analysis of the upper limb lifting movement data was made based on the CNN model. The accuracy of the obtained results indicated that the most significant segment in the classification is a marker paced in the middle of arm (MPH marker). For this study purposes—the two and three class classification have been checked. The proposed methodology has the potential to be used for functional analysis of movement, which can be base for research on model for detailed and accurate monitoring of functional status after stroke, and for qualification for rehabilitation, and for tracking ADLs progression after stroke.

The presented method can be extended to give the ranges of classification accuracy (or different metric) based on multiple runs of proposed training and validation in classification tasks with different dataset splitting. This can produce wider information about influence of particularly upper limb segment to lifting object movement.

Main limitation of this study is that the analysed data is rather small for the CNN models. In the future this model should be checked on bigger dataset. Next limitation concerns its rather expensive implementation. The system requires installation in the laboratory and is therefore not mobile. The presented method in the future should be checked on data obtained from low-cost motion capture systems based on inertial sensors (Held et al., 2018; Pérez et al., 2010) or depth cameras (Chakraborty et al., 2020; Dolatabadi, Taati & Mihailidis, 2017; Bei et al., 2018).

##  Supplemental Information

10.7717/peerj.10124/supp-1 Supplemental Information 1 Marker trajectories Upper body markers trajectores recorded by OptiTrack motion capture system.

## Additional Information and Declarations

### Data Availability

The following information was supplied regarding data availability: The raw measurments are available in a Supplemental File.
